# Feeling right is feeling good: psychological well-being and emotional fit with culture in autonomy- versus relatedness-promoting situations

**DOI:** 10.3389/fpsyg.2015.00630

**Published:** 2015-05-19

**Authors:** Jozefien De Leersnyder, Heejung Kim, Batja Mesquita

**Affiliations:** ^1^Center for Social and Cultural Psychology, Department of Psychology, University of LeuvenLeuven, Belgium; ^2^Department of Psychological and Brain Sciences, University of California at Santa BarbaraSanta Barbara, CA, USA

**Keywords:** Emotion, culture, well-being, emotional fit, cultural fit, autonomy, relatedness, psychological well-being

## Abstract

The current research tested the idea that it is the cultural fit of emotions, rather than certain emotions *per se*, that predicts psychological well-being. We reasoned that emotional fit in the domains of life that afford the realization of central cultural mandates would be particularly important to psychological well-being. We tested this hypothesis with samples from three cultural contexts that are known to differ with respect to their main cultural mandates: a European American (*N* = 30), a Korean (*N* = 80), and a Belgian sample (*N* = 266). Cultural fit was measured by comparing an individual’s patterns of emotions to the average cultural pattern for the same type of situation on the Emotional Patterns Questionnaire (De Leersnyder et al., 2011). Consistent with our hypothesis, we found evidence for “universality without uniformity”: in each sample, psychological well-being was associated with emotional fit in the domain that was key to the cultural mandate. However, cultures varied with regard to the particular domain involved. Psychological well-being was predicted by emotional fit (a) in autonomy-promoting situations at work in the U.S., (b) in relatedness-promoting situations at home in Korea, and (c) in both autonomy-promoting and relatedness-promoting situations in Belgium. These findings show that the experience of culturally appropriate patterns of emotions contributes to psychological well-being. One interpretation is that experiencing appropriate emotions is itself a realization of the cultural mandates.

## Introduction

Within a given culture, people tend to experience similar patterns of emotions, given the same situation. This becomes particularly clear when contrasting the emotional patterns from people from different cultures. Take for instance a student at an end-of-the-year ceremony who receives an applause for being the ‘best student of the year’: a European American student would typically experience pride and excitement in this situation; a typical Belgian student would experience embarrassment in addition to pride and excitement. The example illustrates that the typical patterns of emotions are culture-specific; a finding that has been confirmed by systematic cross-cultural studies on emotion (Kitayama et al., 2006; De Leersnyder et al., 2011, 2013, submitted).

Culture-specific patterns of emotions reflect cultural values and priorities (Mesquita, 2003; Mesquita and Leu, 2007). The emotions of the typical European American student in the example above (pride and excitement) emphasize the value of individual success and autonomy (e.g., Lazarus, 1991; Tracy and Robins, 2004); the emotions of the typical Belgian student (embarrassment in addition to pride and excitement) reflect a concern with others and the relationship as well as with autonomy (e.g., Parrott and Smith, 1991; Keltner and Buswell, 1997). Seen this way, the extent to which an individual’s emotions are similar to the culture’s average emotional pattern in the situation reflects his or her adoption of cultural values and priorities. Cultural fit, or having the typical or ‘right’ emotions, is tantamount to meeting ‘the cultural mandate’ (Kitayama et al., 2010).

In the current research, we test the idea that emotional fit with culture (EFC) is linked to psychological well-being – i.e., being satisfied with oneself, having positive feelings, accepting one’s body and having no symptoms of depression (e.g., Power et al., 1999). We postulate that people who feel the culturally typical emotions “achieve well-being and health through actualizing the respective cultural mandates” (Kitayama et al., 2010, p. 1), particularly in situations that are crucial for the realization of these mandates. We will test this hypothesis for three contexts that have been associated with different cultural mandates: the United States (Study 1), Korea (Study 2), and Belgium (Study 3).

### Cultural Mandates and Emotions

Cultural mandates differ along the dimension of independence and interdependence (D’Andrade, 1984; Markus and Kitayama, 1991a, 2003; Fiske et al., 1998). In ‘independent’ cultural contexts, such as European American and Western European (e.g., Belgian) contexts, the primary mandate is to be autonomous, distinct, and separate from others. In contrast, in ‘interdependent’ cultural contexts such as Korea, the primary mandate is to be related, embedded, and connected to others (e.g., Markus and Kitayama, 1991a, 2003; Kim and Markus, 1999; Rothbaum et al., 2000).

People within these contexts may realize the cultural mandates in different ways. One way is by engaging in everyday cultural practices that instantiate the cultural mandate; examples are sleeping arrangements for infants that either emphasize autonomy (sleeping alone) or relatedness (sleeping with the parents; Shweder, 1991; Morelli et al., 1992), award ceremonies that highlight autonomy (D’Andrade, 1984), and politeness rituals that underscore relatedness (Ide, 1998; Burdelski, 2010). Another way to realize cultural mandates is by engaging in one of the many psychological tendencies that ratify autonomy and/or relatedness; examples are self-enhancing strategies that affirm autonomy (Heine et al., 1999), adopting a third-person perspective that underscore relatedness (Cohen et al., 2007), and – last but not least – experiencing and expressing emotions that highlight either autonomy or relatedness (Kitayama et al., 2000; Mesquita and Karasawa, 2002; Mesquita, 2003, 2010; Mesquita and Leu, 2007; Mesquita et al., 2014; De Leersnyder et al., submitted). Referring to the earlier example of the student at the end-of-the-year ceremony: Feeling pride emphasizes the value of the student in the achievement domain, and thus is a realization of the cultural mandate of autonomy; in contrast, feeling embarrassment is proof that the evaluation by others in the social network is salient to the student, and is an instantiation of relatedness.

By experiencing certain types of emotions people may thus realize a given cultural mandate. In this research, we capitalize on the distinction between autonomy-promoting and relatedness-promoting emotions. This dimension has been found to structure the domain of emotional experience across diverse cultural contexts. (Kitayama and Markus, 1990; Kitayama, Markus and Negishi, 1989 as cited in Markus and Kitayama, 1991b; Kitayama et al., 2000, 2006, see also De Leersnyder et al., submitted). Moreover, it is useful in describing cultural differences in emotional experience: Autonomy-promoting emotions are more intense and prevalent in independent cultural context, and relatedness-promoting emotions are more intense and prevalent in interdependent cultural contexts (Briggs, 1970; Frijda and Mesquita, 1994; Markus and Kitayama, 1994; Mesquita and Karasawa, 2002; Cole et al., 2006; Kitayama et al., 2006; Boiger et al., 2013b, 2014).

The current research goes beyond existing research by focusing on *individual differences* in *EFC*. This means that, rather than focusing on cultural differences in mean level intensities of autonomy-promoting and relatedness-promoting emotions, we will examine individuals’ cultural fit of a wide range of different emotions in situations that are typically associated either with autonomy or relatedness-promoting emotions; we will call these situations autonomy-promoting and relatedness-promoting, respectively. For instance, the situation we described earlier of the student receiving an applause, can be considered a ‘positive autonomy-promoting emotional situation’ in both European American and Belgian contexts, because pride was the primary emotion. We expect that fit in situations that are central to the cultural mandate, for example autonomy-promoting situations in European American contexts, will play a positive role in an individual’s adjustment.

### Emotional Fit with Culture

To establish emotional fit, we consider the *patterns* of emotional experience; i.e., the pattern of co-occurring emotions. Patterns of emotional experience provide a more comprehensive picture of individuals’ interpretations of the situation than could be obtained by looking at single emotions alone, and culture-specificity of emotional experience is also better captured by the respective patterns of emotional experience. This is illustrated by the example about the student who receives applause, in the beginning of the section “Introduction.” In this example, the most intense emotion for the European American and the Belgian student alike was pride, yet the Belgian student also experienced embarrassment – an emotion that was absent from the European American pattern. The patterns of co-occurring emotions (pride only versus pride and embarrassment) describe the meaning of the event more accurately than would the most intense emotion by itself. Therefore, fit with the cultural mandate is best inferred from the pattern of emotion.

We previously designed a measure of emotional co-occurrence: the Emotional Pattern Questionnaire (EPQ; De Leersnyder et al., 2011). Adopting the EPQ, we have repeatedly found that people fit the average emotional patters of the same culture better than those of another culture (De Leersnyder et al., submitted). We have also found a consistently lower fit of minority than majority members to the average majority emotional pattern, with individual minority member’s fit predicted by the level of exposure to the majority culture (De Leersnyder et al., 2011, 2013; Jasini et al., manuscript in preparation). One interpretation of the latter finding is that, over time, immigrants learn to meet the new cultural mandate.

We have some first evidence that an individual’s emotional fit to the cultural average predicts positive outcomes. Using the same EPQ, we found that individuals’ EFC in *relatedness-promoting situations* predicted their level of *relational well-being* – that is, their satisfaction with social relationships and social support – even after controlling for other types of well-being (De Leersnyder et al., 2014). We replicated this finding in the United States, Belgium, and Korea. The finding was limited to situations that were relatedness-promoting; we did not find any relationship between fit in other situations (i.e., self-focused, autonomy-promoting situations) and relational well-being. Yet, for the Korean sample, we found that cultural fit in relatedness-promoting situations was not only associated with higher relational well-being, but also with higher *psychological well-being*. In the current article, we further investigate the association of emotional fit and psychological well-being across the same three cultural groups^1^.

### Emotional Fit with Culture in Focal Domains and Psychological Well-Being

The central hypothesis in the current research is that cultural fit in emotions is conducive to *psychological* well-being. Following earlier definitions (e.g., Power et al., 1999), we conceptualize psychological well-being as being satisfied with oneself, having positive feelings, accepting one’s body and having no symptoms of depression. In contrast to relational well-being that refers to ‘having good relationships’ with other people, psychological well-being refers to ‘being satisfied with yourself as a person.’ Different from relational well-being, which is cross-culturally predicted by emotional fit in relatedness-promoting situations (De Leersnyder et al., 2014), we expect that psychological well-being will be uniquely associated with emotional fit in situations that are central to the cultural mandate. It is these situations that define personhood within the culture. Specifically, we expected that psychological well-being is predicted by the fit of relatedness-promoting situations for interdependent cultures (e.g., Korea), and of autonomy-promoting situations for independent cultures (e.g., United States, Belgium). Moreover, we expected that fulfillment of some cultural mandates may be situation-specific. As we will detail below, work contexts may be better suited to meet cultural mandates of autonomy that require a person, among other things, to “be a strong leader” and to “take initiative to achieve personal success” (Kitayama and Imada, 2010), whereas home contexts may be better suited to meet cultural mandates of relatedness that require a person to “conform and to be obedient,” and to “achieve social harmony.” Across cultures, different contexts and situations may thus afford the realization of the culture’s cultural mandate, which we expect to be associated with psychological well-being.

We thus expect “universalism without uniformity” (Shweder and Sullivan, 1993): Universally, we expect psychological well-being to be associated with emotional fit in domains that are most central to the cultural mandate; however, we expect that the specific domains will vary by culture. This hypothesis is consistent with a growing body of literature suggesting that fulfilling the cultural mandates of autonomy (such as maintaining high self-esteem) may be most conducive to psychological well-being in independent cultural contexts, whereas fulfilling the cultural mandates of relatedness (such as having harmonious social relationships) may be most conducive to psychological well-being in interdependent cultural contexts (Kwan et al., 1997; Kang et al., 2003; Kitayama et al., 2010). In the current research, we extend this hypothesis to the emotional realm.

Two studies provided some first support for the idea that psychological well-being is particularly associated with *emotional* fit in domains that are central to the cultural mandate (Kitayama et al., 2006; Tsai et al., 2006). In an experience sampling study, European American college students’ general positive feelings (e.g., feeling happy) were predicted by the intensity ratings of positive autonomy-promoting emotions (e.g., pride), whereas Japanese students’ general positive feelings were predicted by the intensity of positive relatedness-promoting emotions (e.g., friendly feelings; Kitayama et al., 2006). In another study with European American and Hong Kong Chinese students, negative psychological well-being (i.c., depressive symptoms) was predicted by the discrepancy between actual emotions and the emotions people “would like to feel” over the course of a week (Tsai et al., 2006), but only with respect to the emotions that were central to the respective cultural mandates (Tsai et al., 2007). Both studies thus provide first support for the hypothesis that emotional fit selectively predicts psychological well-being in different cultures.

However, our understanding of the relationship between EFC and psychological well-being is still fairly limited. Firstly, both studies *inferred* EFC rather than measured the fit. Secondly, both studies predicted well-being from intensity ratings of averaged emotion scales and did not consider the patterning of emotions. Finally, both studies disregarded the situational origin of the emotion intensity ratings when using these intensity ratings to predict well-being. To gain a full understanding of the processes, the current research will *measure, actual, fit* with cultural patterns of emotions by correlating an individual’s pattern of emotion intensities to the culture’s average pattern of emotion intensities for a particular type of situation (cfr., De Leersnyder et al., 2011), thereby focusing on the *patterning* of emotions instead of on their mean levels of intensity. Finally, the current research formulates more precise, a priori hypotheses about the relevant situations of emotional fit in different cultures, defining both the types of primary emotions (autonomy versus relatedness-promoting) and the contexts (home, work) involved. Next, we will lay out which situations are central to the respective cultural mandates of the samples included.

#### European American Cultural Context

As outlined above, independent cultural contexts, such as the European American, highlight the cultural mandates of autonomy. More specifically, European American cultural contexts endorse a pure form of autonomy, where standing out among others, and achieving personal success are important (Schwartz and Ros, 1995; Heine et al., 1999; Kitayama et al., 2009; Stephens et al., 2009; Oishi, 2010; Boiger et al., 2013a; Boiger, unpublished doctoral dissertation). Important cultural mandates are “expressing one’s unique self,” “being a strong leader,” “taking initiative to achieve a personal success” and “being in charge and under control” (Kitayama and Imada, 2010). These cultural mandates are best realized in situations that evoke autonomy-promoting emotions; moreover, work contexts can be expected to be more conducive to the cultural mandate than home contexts. In support of the latter, research in European American contexts has established strong boundaries between work and home contexts, with work contexts stressing competitive autonomy, often at the expense of relatedness (Sanchez-Burks and Uhlmann, 2014). Taken together, we expect that cultural fit of emotions in European American contexts will be most predictive of psychological well-being in *autonomy-promoting situations at work*.

#### Korean Cultural Context

Interdependent cultural contexts, such as the Korean context, highlight the cultural mandates of relatedness (Markus and Kitayama, 1991a; Rothbaum et al., 2000; Oyserman et al., 2002). Important cultural goals are “conforming and being obedient,” “being similar to others,” “following social norms and fitting-in,” and “achieving social harmony” (Kitayama and Imada, 2010). It has been argued that these cultural mandates are primarily realized in the context of close in-groups (Markus and Kitayama, 1991a), with a particular emphasis on the family within Korean cultural contexts (Neuliep, 2011). Therefore, we expect that emotional fit in Korean cultural contexts will be most predictive of psychological well-being in *relatedness-promoting situations in the family context*.

#### Belgian Contexts

Although Western European cultures, such as Belgium, are considered to be independent, they endorse a less pure form of independence than European American cultural contexts (Kitayama et al., 2009; van den Bos et al., 2010). The European form of independence stresses “the integrity of the individual within a social network of equal rights” (Boiger, unpublished doctoral dissertation; p. 84) and thus emphasizes that there is room for autonomy as far as it does not jeopardize a person’s relatedness within a social network (e.g., Schwartz and Ros, 1995; Boiger et al., 2013a). In the Belgian context, the mandate to be autonomous thus goes hand in hand with the mandate to be related to others in a social network. Therefore, psychological well-being is expected to be *primarily* associated with cultural fit in situations that elicit autonomy-promoting emotions, yet *also* with cultural fit in situations that elicit relatedness-promoting emotions. Moreover, and consistent with the domain-specificity of the opportunities to realize the cultural mandates, we hypothesize that in the Belgian context, psychological well-being will be linked to emotional fit in *autonomy-promoting situations at work* as well as to emotional fit in *relatedness-promoting situations with family and with friends*.

### Overview of the Current Research

In three studies, we test the hypothesis that cultural fit predicts psychological well-being in situations and contexts that are central to the cultural mandate. We expect psychological well-being to be predicted by (i) autonomy-promoting situations at work for European Americans (Study1); (ii) relatedness-promoting situations at home for Koreans (Study 2); and (iii) both autonomy-promoting situations at work and relatedness-promoting situations at home and with friends for Belgians (Study 3).

## General Method

### Materials

#### Cultural Fit in Emotions

To measure EFC, we adopted the EPQ (De Leersnyder et al., 2011). The EPQ has been validated in previous research on EFC (e.g., De Leersnyder et al., 2011, 2013) and is particularly suited to investigate fit in the outlined types of culturally focal situations. In fact, participants in the EPQ respond to a set of questions after reading a prompt that is defined by valence (positive, negative), social context (Family, Work/School, Friends), and the autonomy versus relatedness-promoting nature of the situation. Prompts for autonomy-promoting situations ask a person to think about an emotional situation that was *“about things that happened to you personally”* and list either positive or negative autonomy-promoting sample emotions that are expected to be most intense in the situation (e.g., pride, on top of the world, superior for positive autonomy-promoting situations); prompts for relatedness-promoting situations ask a person to think about an emotional situation that was *“about your relationship with others”* and list positive or negative relatedness-promoting sample emotions (e.g., ashamed, guilty, indebted for negative relatedness-promoting situations). After reading the prompt, participants were asked to describe (in writing) a situation from their own recent past that matched the prompt. For instance, the prompt for a positive autonomy-promoting situation at work/school read as follows:

“Sometimes, people find themselves in situations that make them feel ***good for themselves*** (for example, superior, proud, top of the world). Please think about an occasion **at work or at school** in which you felt **good for yourself** (for example, superior, proud, top of the world). Please take your time to remember this situation. Please describe the situation briefly. Provide as much detail as needed for somebody to understand why you felt that way in this situation.”

After describing the situation, participants rated the intensity of their emotions in that situation on a set of emotion scales (20 in Study 1 and 2, and 34 in Study 3) that cover the domain of emotional experience (as in De Leersnyder et al., 2011). The intensity ratings (1 = *totally not* – 7 = *extremely*) of the full set of emotions constitute an individual’s emotional pattern for a specific type of situation. We calculated each participant’s *EFC* by (i) calculating the cultural sample’s average emotion pattern for each type of situation, and (ii) running profile correlations between each individual’s pattern and the average cultural pattern for the corresponding situation. In this way, the EPQ captures emotional fit for both positive and negative *autonomy-promoting* and *relatedness promoting* situations across different social contexts such as at home, at work or school, and with friends.

As in our previous studies (e.g., De Leersnyder et al., 2011, 2014), we made use of profile correlations (i.e., Pearson correlations across the individual’s and the sample’s average emotional profiles) to capture the fit. Profile correlations have the advantage that they (i) take into account the similarity across a whole set of emotions; (ii) capture the idea of emotional patterns (i.e., the relative intensities of different emotions); and (iii) are not prone to individual differences in scale use. Before establishing the correlations, we excluded emotion items from the pattern if there was no within sample agreement on their meaning [as suggested by low or cross-loadings on a (Varimax rotated) solution of a Principal Component Analysis; cfr. infra for more details on these analyses]. Furthermore, each participant’s own scores were omitted from the average cultural sample’s pattern to which they were compared. The correlation coefficients were Fisher *z*-transformed to achieve a linear distribution of the data (a requirement for the statistical techniques used).

Given that the results for positive and negative situations were similar (see Supplementary Table A2), we collapsed the fit scores across negative and positive situations. Thus we obtained one fit score for autonomy-promoting situations and one fit score for relatedness-promoting situations.

#### Psychological Well-Being

We measured psychological well-being with either the long (Studies 1 and 2) or the short (Study 3) version of the World Health Organization’s Quality of Life Questionnaire (WHOQOL Group, 1995; Power et al., 1999; Skevington et al., 2004). The WHOQOL Group (1995) captures relevant well-being domains across different cultures (e.g., Power et al., 1999), and is suitable for non-clinical samples. Its psychological well-being subscale covers several aspects of psychological well-being, thereby providing a more thorough estimation of the construct than many other scales: ‘positive feelings,’ ‘thinking and concentration,’ ‘self-esteem,’ ‘body image,’ ‘spirituality’ (i.e., meaning in life) and ‘negative feelings’ (reverse coded).

Another advantage is that the WHOQOL Group (1995) captures more well-being domains than only psychological well-being. In fact, both the long and the short version of the WHOQOL cover 24 facets that cluster into four broad domains of well-being: psychological, physical, environmental, and relational well-being. In the current research, all well-being domains that do not refer to psychological well-being (i.e., relational, environmental, and physical well-being) were combined in an *Overall Quality of Life index.* This index was used as a control variable when testing the link between emotional fit and psychological well-being, which allowed us to investigate the *net contribution* of emotional fit to the prediction of psychological well-being above and beyond other indices of well-being (for a similar approach see Carton et al., 1999 and De Leersnyder et al., 2014). Each time, higher domain scores (20-point scale in the long version; five-point scale in the short version) indicate higher well-being.

#### Demographic Variables and Informed Consent

Before the start of each study, participants received, read, and signed an informed consent about the study (studies approved by the University of California at Santa Barbara Human Subjects Committee). At the end of each study, all participants completed demographic questions for age, gender, and social class. Since these demographic variables are known to be associated with psychological well-being (e.g., González Gutiérrez et al., 2005; Akhtar-Danesh and Landeen, 2007) we will also control for these variables when testing our hypotheses (see Supplementary Table A1, for an overview of the raw correlations between each of the control variables and the variables of interest in the current research.

### Data-Analysis

In order to test whether people’s psychological well-being is associated with their EFC in either autonomy-promoting or relatedness-promoting situations at home or at work, we conducted a linear regression analysis (see **Table 1**) in which we predicted Psychological Well-being from EFC after controlling for (i) the Context of the emotional fit measurements (Step 1), Demographic variables (Step 2), and Overall Quality of Life index (Step 3). In the fourth step, we always tested the main effects of EFC in autonomy and relatedness-promoting situations and, in the fifth step we finally tested our hypothesis in the form of interactions between EFC and the Context in which fit was measured.

**Table 1:** Results of hierarchical linear regressions predicting psychological well-being from emotional fit with culture in autonomy- and relatednesspromoting situations in home, work and friend contexts.

Panel A: Study 1: Euro-American cultural context			Panel B: Study 2: Korean cultural context			Panel C: Study 3: Belgian cultural context		
Predictor	Δ*R*^2^	β^a^	Predictor	Δ*R*^2^	β^a^	Predictor	Δ*R*^2^	β^a^
*Step 1*	0.001 (0.001)		*Step 1*	0.047^†^(0.047^†^)		*Step 1*	0.006	
Work-dum		0.040 (.015)	Work-dum		0.180 (0.180)^*^	Work-dum		0.049
						Friends-dum		0.057
*Step 2*	0.245(0.245)		*Step 2*	0.127(0.127)		*Step 2*	0.016	
Age		0.097 (0.132)	Age		0.144 (0.155)	Age		0.153
Gender		0.199 (0.242)	Gender		0.004 (0.002)	Gender		0.071
Class		0.211 (0.217)	Edu duml		0.028 (0.025)	Edu Mother		0.004
			Edu dum2		0.037 (0.049)	Edu Father		0.025
*Step 3*	0.409^***^(0.409^***^)		*Step 3*	0.449^***^(0.449^***^)		*Step 3*	0.303^***^	
Overall QOL		0.770^***^(0.709^***^)	Overall QOL		^***^0.695 (0.694^***^)	Overall QOL		0.563^***^
*Step 4*	0.407 (0.000)		*Step 4*	0.007 (0.005)		*Step 4*	0.037^***^	
EFC Auto		-0.321 (0.444^*^)	EFC Auto		0.009	EFC Auto		0.138^*^
EFC Rela		-0.247	**EFC Rela**		0.260^†^(0.264^*^)	EFC Rela		**0.103**
*Step 5*	0.144^***^(0.134^***^)		*Step 5*	0.024^††^(0.024^*^)		*Step 5*	0.010	
						Work-dum X EFC Auto		
** Work-dum X EFC Auto**		0.511^***^(0.614^***^)	Work-dum X EFC Auto		0.036	Work-dum X EFC Rela		
Work-dum X EFC Rela		0.141	** Work-dum X EFC Rela**		0.253^†^(0.247^*^)	Friends-dum X EFC Auto		
						Friends-dum X EFC Rela		
Total *R*^2^	0.815^***^(0.789^***^)		Total *R*^2^	0.654^***^(0.606^***^)		Total *R*^2^	0.363^***^	

*Hypothesized associations appear in bold. Work-dum, Dummy variable representing Work Contexts; Friends-dum, Dummy variable representing Friends Contexts; Family Context is always the reference category; Edu dum1, dummy variable representing tertiary educational level; Edu dum2, dummy variable representing PhD educational level; Edu Mother, educational level mother; Edu Father, educational level father; Overall QOL index, Overall Quality of Life index; EFC Auto, Emotional Fit with Culture in Autonomy-promoting situations; EFC Rela, Emotional Fit with Culture in Relatedness-promoting situations*.

*^a^The βs presented here are the ones from the final regression model (i.e., the latest step that significantly contributed to the explained variance). The values in between brackets for Study 1 are those for the additionally tested regression model in which we only included Emotional fit in autonomy-promoting situations. The values in between brackets for Study 2 are those for the additionally tested regression model in which we only included Emotional fit in relatednesspromoting situations*.

^††^*p* < 0.15, †*p* < 0.10, ^*^p ≤ 0.05, ^**^p ≤ 0.01, ^***^p ≤ 0.001.

The Context variable was always dummy-coded, with the Family context serving as the reference category. In Studies 1 and 2 we only included Family and Work/School contexts resulting in one dummy variable referring to the Work context: Work-dummy (*0 = Family, 1 = Work/School).* In Study 3, we included a Friends contexts in addition to Family and Work/School contexts, resulting in one Work-dummy (*0 = Family, 1 = Work/School, 0 = Friends)* and one Friends-dummy (*0 = Family, 0 = Work/School, 1 = Friends).* Therefore, the regression coefficients of main effects qualified by interactions pertain to the effects of EFC in Family contexts, and the regression coefficients of interaction terms between EFC and the Work-dummy or Friends-dummy pertain to the effects of EFC in Work contexts and Friends contexts, respectively.

In each study, we excluded participants when the valence of their self-reported situations did not match the valence of the prompt [Study 1, *n* = 3 (9%); Study 2, *n* = 5 (6%); Study 3, *n* = 9 (3%)]. In addition, some participants could not be included in the analyses due to missing data for either the well-being questionnaire or the EPQ prompts [Study 1, *n* = 1, Study 2, *n* = 3, Study 3, *n* = 16].

## Study 1

### Participants and Procedure

Participants were 30 European Americans from a community sample [66.6% female; *M*_age = 38.5 years (SD_age = 14); *Median_*_social_class_ = 3, corresponding to *solidly middle class*, on a scale from 1 = *working class* – 5 = *upper class*]. Participants were recruited in public places, such as malls, and received $10 for their participation.

All participants completed four versions of the EPQ: two were autonomy-promoting and two were relatedness-promoting (one positive and one negative for each). Context was thus the only between-subjects factor in this study: Each participant completed all prompts with respect to the same context (Family *n* = 17; Work/school *n* = 13). The order of the prompts was counterbalanced, but there were no order effects. Furthermore, a Principal Component Analysis (with Varimax rotation) on the emotion items (interested, strong, proud, close, respect, helpful, guilty, ashamed, afraid, indebt, worthless, embarrassed, upset, irritable, bored, ill feelings, resigned, jealous, relying, surprised) yielded a clear three-factor structure that explained 60% of variance in the data. This means that all emotion items were interpreted in a similar way within this sample, allowing us to retain all emotion items when calculating emotional fit with the cultural sample’s average. Participants completed the long version of the WHO Quality of Life scale. *Psychological well-being* was calculated on the basis of four facets because the reliability analysis indicated that the Cronbach’s alpha improved by excluding the facets ‘spirituality’ and ‘thinking and concentration’ from the scale; (*Psychological well-being* α_facets_ = 0.87; *M* = 13.91 SD = 3.21; *Overall Quality of Life index* α_facets_ = 0.84; *M* = 15.1 (SD = 2.03).

### Results and Discussion

As hypothesized, the regression model including the interaction terms between EFC and Context (i.e., Work-dummy) was significant and predicted most variance in Psychological Well-being (Step 5: Δ*R*^2^ = 0.114, *p* = 0.010; see **Table 1**, panel A, for the full results). As expected, only the effect of EFC in *autonomy-promoting situations at school or work* (interaction term) was significant, and it was positively associated with European Americans’ psychological well-being (β_EFC-Autonomy_ = -0.321, *p* = 0.103; β_Work-dummyX_ _EFC_Autonomy_ = 0.511, *p* = 0.010)^2^. When removing the non-significant EFC predictors from this model, the results were more pronounced, with the negative coefficient of EFC in Autonomy-promoting situations in Family contexts (main effect) becoming significant (see **Table 1**, panel A, in between brackets; Step 5: Δ*R*^2^ = 0.134, *p* ≤ 0.001; β_EFC-Autonomy_ = -0.440, *p* = 0.019; β_Work-dummy X EFC_Autonomy_ = 0.614, *p* = 0.001). Simple slopes analyses on the basis of this latter model (i) confirmed our hypothesis that European American’s psychological well-being was positively associated with EFC in Autonomy-promoting situations in Work contexts (simple slope, *B* = 3.684, SE = 1.455, *p* = 0.020), yet also revealed that European Americans’ EFC in Autonomy-promoting situations in Family contexts was negatively (instead of not) associated with their psychological well-being (simple slope: *B* = -4.134, SE = 1.619, *p* = 0.019). Follow-up analyses with the four different indices of emotional fit (see Supplementary Table A2, panel A) revealed that this latter effect was driven by the effect of EFC in negative autonomy-promoting situations, which center around irritation and ill-feelings.

Despite its small sample size, Study 1 provides first support for our hypothesis that people’s psychological well-being is linked to their emotional fit in situations that are central to the cultural mandate. Indeed, European Americans reported fewer depressive symptoms, more positive feelings about their lives, more self-esteem, etc., as their patterns of emotions fitted those of other European Americans in autonomy-promoting situations at work. To ensure that these findings were specific to emotional fit in culturally focal domains and not due to high levels of autonomy-promoting emotions (which may be linked to self-esteem), Study 2 tested our hypothesis in a sample of Koreans for whom the culturally focal domains do not include situations that foster autonomy-promoting emotions, but rather foster relatedness-promoting emotions.

## Study 2

### Participants and Procedure

Participants were 75 Koreans from a community sample (60% female; *M*_age = 28 years; SD_age = 4.25). As an index of socioeconomic status, participants reported their highest degree of education [dummy-coded as ‘Edu dum1’ = college (*n* = 41); ‘Edu dum2’ = graduate school (*n* = 9); with “reference group” = high school (*n* = 26)]. Participants received ₩10.000 (about 10 dollars) for completing the questionnaires and were recruited through a Christian mega-church; these churches are common, given that Christianity is widely practiced in Korea (37%). The design and materials were the same as those used in Study 1. Each participant completed all prompts with respect to the same context (Family *n* = 35 Work/school *n* = 37). Again, there were no order-effects and we collapsed the emotional fit scores into one score for EFC in Autonomy-promoting situations and one score for EFC in Relatedness-promoting situations. The Principal Component Analysis (with Varimax rotation) on the emotion data explained 65% of the variance with a three-factor structure on which all but three items (embarrassed, afraid, surprise) loaded well. Consequently, these three items were omitted from our calculations of EFC. As in Study 1, participants completed the long version of the WHO Quality of Life scale (*Psychological well-being* α _facets_ = 0.71; *M* = 13.94 (SD = 2.02; *Overall, Quality of Life* α_facets_ = 0.90; *M* = 14.40 (SD = 1.79).

### Results and Discussion

We followed the exact same analytic strategy as in Study 1. The expected model including the interaction terms between EFC and the Context of the situation did not reach significance above and beyond the control variables (Step 5: Δ*R*^2^ = 0.024, *p* = 0.147)^3^. Yet, an inspection of the regression coefficients revealed that the pattern of results was in line with our expectations: a main effect of EFC in Relatedness-promoting situations (β = 0.260, *p* = 0.054) that was qualified by the interaction between the Work-dummy and EFC in Relatedness-promoting situations (β = -0.253, *p* = 0.054); no effects pertaining to EFC in Autonomy-promoting contexts were significant. When removing these latter, non-significant effects from our model, Step 5 became significant (see **Table 1**, panel B, between brackets; Step 5: Δ*R*^2^ = 0.024 *p* = 0.047; β_EFC_Relatedness_ = 0.264, *p* = 0.038; β_Work-dummy X EFC_Relatedness_ = -0.247, *p* = 0.047. A simple slopes analysis on the basis of this latter regression analysis further confirmed our hypothesis: Korean’s psychological well-being was associated with their EFC in Relatedness-promoting situations in Family contexts (simple slope, *B* = 1.998, SE = 0.94, *p* = 0.038), but not with EFC in Relatedness-promoting situations in Work contexts (simple slope, *B* = -0.566, SE = 0.85, *p* = 0.510).

Study 2 thus provides further evidence for the idea that EFC is linked to psychological well-being, but only in culturally focal domains. In Study 3, we again tested this hypothesis in the Belgian cultural context where the cultural mandate is to be autonomous without jeopardizing relatedness.

## Study 3

### Participants and Procedure

Two-hundred-forty-two psychology freshmen from the University of Leuven (Belgium) participated in this study (83% female; *M*_age_ = 18.82; SD = 1.87; *M*_education mother_ = 3.58, SD = 0.59*; M*_education father_ = 3.57, SD = 0.70). Students participated in this research for course credit.

In the version of the EPQ that we used in this study, participants rated their emotional experience on 34 (instead of 20) items. In addition to the Family context and the Work/School context, the prompts of the EPQ now also included a Friends context. Different from studies 1 and 2, each student completed the EPQ for two different situations, similar in Valence (*n* positive = 116; *n* negative = 126) and Relationship Context (*n* family context = 82; *n* work/school context = 80; *n* friend context = 82), yet one pertaining to an autonomy-promoting situation, the other pertaining to a relatedness-promoting situation. A Principal Component Analysis (with Varimax rotation, explaining 67 and 62% of the variance for emotion data in the first and the second situation, respectively) revealed a clear four factor structure reflecting positive autonomy-promoting, positive relatedness-promoting, negative autonomy-promoting and negative relatedness-promoting emotions. Four items did not load on a single factor (bored, jealous, feeling resigned, and feeling pity) and were omitted from the emotional fit calculations.

Due to time constraints, students completed the short version of the WHO Quality Of Life Questioannire – i.e., the WHOQOL BREF (Skevington et al., 2004). Although this questionnaire has been successfully used with Belgian students (Baumann et al., 2011), other authors have argued to be cautious about its factor structure (Theuns et al., 2010). Therefore, we first conducted a Principal Component Analysis on the 24 items of the WHOQOL BREF. A solution with six factors yielded interpretable factors and explained 57% of the variance in the data. Four factors were similar to those intended by the WHO and referred to psychological, physical, environmental, and relational well-being. However, two additional factors emerged, one clustering three items referring to transportation and finances, the other clustering the items ‘capacity for work,’ ‘being able to perform daily living activities,’ ‘being able to concentrate,’ and ‘feeling that your life is meaningful.’ These two latter items were originally proposed to be items of psychological well-being scale; yet, as they loaded on a different scale, we did not include them in our construct of psychological well-being. The other four items (‘positive feelings,’ ‘self-esteem,’ ‘accepting your body,’ and ‘negative feelings’; reverse coded) formed a reliable *Psychological Well-being* scale (α = 0.75; *M* = 3.57 (SD = 0.60). As in studies 1 and 2, we calculated an *Overall, Quality of Life index* including all items except those constituting the psychological well-being scale [α = 0.83; *M* = 3.84 (SD = 0.43)].

### Results and Discussion

To test whether Belgians’ psychological well-being was associated with their EFC in both autonomy and relatedness-promoting situations, we conducted a regression analysis that was the same as in Studies 1 and 2, except that we now included one dummy variable pertaining to Work contexts and one pertaining to Friends contexts; again Family context served as the reference category. The analysis revealed that a model including the main effects of EFC explained most variance in participants’ psychological well-being above and beyond the control variables (step 4: Δ*R*^2^ = 0.037 *p* = 0.002); the expected interaction effects did not significantly contribute to the model (step 5: Δ*R*^2^ = 0.010 *p* = 0.454). However, in line with the prediction that Belgian cultural contexts are characterized by a less extreme form of independence and that, therefore, the cultural mandate of autonomy goes hand in hand with the mandate of relatedness, our results indicated that Belgians’ psychological well-being was positively associated with their EFC in Autonomy-promoting situations (β_EFC_Autonomy_ = 0.138, *p* = 0.018), and marginally significantly with EFC in Relatedness-promoting situations (β_EFC_Relatedness_ = 0.103, *p* = 0.080; see **Table 1**, panel C)^4^. This latter effect became significant when controlling for the Valence of the situation, although there were no significant interaction effects between Valence and EFC (see Supplementary Table A2, panel C).

## General Discussion

Cultural fit of emotions is associated with psychological well-being, yet only when the fit occurs in domains that are central to the realization of the respective cultural mandates. For instance, we found that European Americans’ psychological well-being was associated with emotional fit in autonomy-promoting situations at work. In these situations, where autonomy-promoting emotions are most intense, the ‘right’ patterning of one’s emotional experiences – i.e., one’s EFC – may reflect to what extent one embodies the cultural mandate of being autonomous in the ‘proper European American way.’ From fitting in emotionally to these situations, European Americans may thus derive feelings of being a competent member of their society, which may boost their positive self-regard and buffer against depression.

In contrast, yet also in line with their cultural mandate, Koreans’ psychological well-being was associated with their EFC in relatedness-promoting situations at home. Finally, we found that whereas Belgians’ psychological well-being was most strongly linked to EFC in autonomy-promoting situations, it was also linked to their EFC in relatedness-promoting situations. Although we had expected that these effects would be qualified by the context of interaction – which they were not – the results are nevertheless in line with the Belgian cultural mandate of egalitarian autonomy that mandates autonomy as long as it not jeopardizes relatedness. These findings thus not only support our main hypothesis, but also further support the idea that both autonomy and relatedness define European (i.e., Belgian) mandates (e.g., Kitayama et al., 2009; Boiger, unpublished doctoral dissertation) and the according models of psychological well-being.

Unexpectedly, the European American study yielded a *negative* association between psychological well-being and emotional fit in autonomy-promoting situations at home; a finding that was completely driven by the fit in negative situations. We had not predicted this association, but it is intuitive nonetheless. Fit with negative autonomy-promoting situations at home means that emotions such as anger, irritation and ill feelings are most intense. It is not surprising that these feelings are associated with negative psychological well-being, even in European American samples. Conflict at home is not desirable, and European American families form no exception to this rule: Having fewer negative autonomy-promoting emotions than average, and thus having lower cultural fit, is a sign of psychological health.

The current research contributes to previous research on the link between emotions and psychological well-being in several ways. First, the current studies actually *measured EFC* rather than inferred it. Consequently, they provide more direct evidence for the link between psychological well-being and EFC than previous studies (Kitayama et al., 2006; Tsai et al., 2006). Second, they go beyond traditional studies by considering the *patterns of co-occurring emotions*, rather than discrete emotions. Indeed, traditional approaches have linked well-being to the *intensity* of culturally appropriate emotions, regardless of the situation in which they occurred. In contrast, the current studies linked the *patterning* of a whole set of emotions to psychological well-being. In a series of *post hoc* analyses, we found that cultural fit predicted participants’ psychological well-being over and above mean intensity levels of autonomy-promoting (e.g., pride and anger) and relatedness-promoting (e.g., closeness and shame) emotions (see Supplementary Material pages 4–7 and Supplementary Table A4). Thus, indices of cultural fit within particular situations were better predictors of psychological well-being than the mean intensity levels of prototypical emotions; a finding that was true across the three studies and across different situations. As such, the current research highlights the benefits of the *cultural fit*, and suggests that the utility of particular emotions is dependent on the specific situational and cultural context.

### Toward a Cultural Psychology of Psychological Well-Being

The current research resonates with a growing body of research that defines psychological well-being in terms of fit with culture. Most of the evidence is indirect, explaining culturally different predictors of well-being from (putative) differences in the cultural mandates. For instance, in a study comparing representative samples of Americans and Japanese (Kitayama et al., 2010), well-being in US contexts was predicted by the level of perceived ‘personal control,’ whereas in Japanese contexts, the perceived absence of ‘relational strain’ was a better predictor of well-being. Personal control helps to realize the cultural mandate of autonomy, whereas the absence of relational strain is instrumental to the cultural mandate of relatedness (see also Kwan et al., 1997; Kang et al., 2003). Thus, culture-congruent psychological processes predicted psychological well-being.

More indirect evidence comes from studies showing that the most prevalent or most valued psychological dimensions best predict well-being in a given culture. For instance, the personality traits that best predicted well-being were the ones shared within a cultural group, such as extraversion in a culture with high levels of extraversion (Fulmer et al., 2010). Likewise, adolescents’ self-esteem was best predicted by a positive self-evaluation in the domain valued most by others in the culture (Becker et al., 2014). Correspondingly, negative well-being has been related to psychological tendencies that violate the cultural mandate. In Hong Kong and Mexico, where the cultural mandate is relatedness, individuals with an avoidant attachment style (associated with high autonomy and low relatedness) experienced more relationship problems than did individuals with the same attachment style in the US (Friedman et al., 2010).

Even more germane to our research are studies on *cultural consonance* (e.g., Dressler, 2012). Cultural consonance refers to “the degree to which individuals approximate, in their own beliefs and behaviors, the prototypes for belief and behaviors encoded in shared cultural models” (Dressler, 2012, p. 2). Methodologically, cultural consonance is measured in a similar way as EFC – i.e., by calculating profile correlations between an individual’s answers to a set of questions in a particular domain and the aggregated set of answers from their own cultural group. Across multiple studies with Brazilians and African Americans, individuals’ cultural consonance in the domains of family life, social support, lifestyle, national identity, and food was found to be associated with lower psychological distress and fewer depressive symptoms (Dressler and Bindon, 2000; Dressler et al., 2007). Moreover, these studies revealed that Brazilians’ consonance in the domain of family life – which is *most central* to the cultural mandate (e.g., DaMatta, 1985) – was *most* predictive of their changes in depressive symptoms, even after controlling for both the cultural fit in other domains and stressful life events (Dressler et al., 2007).

In all these studies, cultural fit of psychological processes rather than the specific psychological phenomena themselves predict psychological well-being. In our research we found that emotional fit particularly in situations that were central to the realization of the respective cultural mandates counted toward psychological well-being. This finding may have important implications for the clinical practice, as it implies that well-being, and conversely ill-being and psychopathology, may be an emergent property of the interaction between mind and culture (Mesquita and Walker, 2003; Ryder et al., 2011).

### Limitations and Avenues for Further Research

The current research is not without its limitations. First, the sample sizes for our studies ranged from very small (Study 1) to medium (Study 3), which may have weakened the power of our regression analyses. However, despite the sample size, the convergence of the results across the three studies is remarkable and strengthens our confidence in the findings. Relatedly, cultural fit of emotions was not established with regard to a *representative* cultural sample. Relatedly, we did not establish cultural fit of emotions with regard to a representative cultural sample. Rather, we established an individual’s emotional fit with regard to the cultural subgroup that is socially relevant to the individual as he or she engages in it on a daily basis. Future research should test whether it is emotional fit with the average patterns of one’s local community or emotional fit with the representative patterns of one’s wider nation that is most closely associated with well-being.

A second limitation of the current studies is that our data are cross-sectional and do not allow us to establish the direction of the link between psychological well-being and EFC. On the one hand, studies have suggested a causal link from better emotional fit to better well-being. For instance, the emotional fit of romantic partners or roommates predicts satisfaction with the relationship 6 months later (Anderson et al., 2003; Gonzaga et al., 2007) and the emotional fit of anxious interaction partners buffers against stress during a following laboratory speech task (Townsend et al., 2013). On the other hand, the *cultural norm hypothesis* on depression proposes that depressive symptoms reduce people’s attention to cultural norms of emotional reactivity, thereby suggesting a causal link from well-being (i.c., depression) to emotional fit (i.c., misfit) with cultural norms (Chentsova-Dutton et al., 2007, 2010). Of course, a feedback loop between the two, with links in two directions, is most likely.

Finally, we did not investigate the precise mechanism through which EFC is associated with psychological well-being. Future research may focus on possible mediators of this link, such as (a) the conscious *distress of not fitting in*, which partially mediated the effects of cultural (mis)fit on depression in the studies by Dressler and colleagues (e.g., Balieiro et al., 2011; see also Townsend et al., 2013), (b) perceived *shared reality,* which socially validates ‘the way people are’ and, as such, boost their sense of epistemic competence and feelings of psychological well-being, as speculated by Fulmer et al. (2010; Hardin and Higgins, 1996), and (c) the *social consequences* of experiencing the ‘right’ emotions (e.g., Keltner and Ha idt, 2001; Szczureck et al., 2012).

Despite these limitations, the current research clearly suggests that psychological well-being is associated with emotional fit in culturally focal domains in which the fit stands for a person’s embodiment of the culture’s mandate in the culturally appropriate ways.

## Conflict of Interest Statement

The authors declare that the research was conducted in the absence of any commercial or financial relationships that could be construed as a potential conflict of interest.

## Footnotes

1 In the current research, we will make use of the same three samples as the ones reported on in De Leersnyder et al. (2014); the analyses are not overlapping, since the outcome variable of interest is different.

2 This pattern of results was highly similar if we did not control for the *Overall QOL Index* (see Supplementary Table A3, panel A for the full results of this analysis).

3 Not controlling for Overall Quality of Life Index, yielded a very similar pattern of results, be it that the interaction effect was even weaker (see Supplementary Table A3, panel B for the full results of this analysis).

4 Not controlling for Overall Quality of Life Index, yielded a similar pattern of results (step with main effects: Δ*R*^2^ = 0.036 *p* = 0.015; β_EFC_Autonomy_ = 0.101, *p* = 0.149; β_EFC_Relatedness_ = 0.136, *p* = 0.056); yet, the overall model was only marginally significantly better than the 0-model (i.e., without any predictors; *F*_8,233_ = 1.748, *p* = 0.089; see Supplementary Table A3, panel C for the full results of this analysis).
