# Supplementary material for: Feeling right is feeling good: psychological well-being and emotional fit with culture in autonomy- versus relatedness-promoting situations
Source: Front Psychol. 2015 May 19;6:630. doi: 10.3389/fpsyg.2015.00630 (PMC4436561; doi:10.3389/fpsyg.2015.00630)
Supplement: Supplementary file 1 [file Table_1.DOCX]

**Online Supplementary Materials
Feeling right is feeling good: psychological well-being and emotional fit with culture in autonomy- versus relatedness-promoting situations**

Table A1. Raw Correlations between Psychological Well-being and each of the other variables included in our analyses.

|  | **Panel A:**  **Study 1: European Americans** | | | | | **Panel B:**  **Study 2: Koreans** | | | **Panel C:**  **Study 3: Belgians** | | | | |
| --- | --- | --- | --- | --- | --- | --- | --- | --- | --- | --- | --- | --- | --- |
|  | | Psychological well-being | | Overall QOL index | | Psychological well-being | | Overall  QOL index | Psychological well-being | | Overall  QOL index | | |
| Age | | | -.240 | | -.083 |  | .221† | -.018 |  | .102 | | -.075 | |
| Gender | | | -.380* | | -.101 |  | .069 | .069 |  | -.035 | | .057 | |
| Socio-Economic Status | | Class | .072 | | .345* | Edu 1  Edu 2 | .110  .255* | -.022  .271* | Edu Mo  Edu Fa | .014  .059 | | .069  .066 | |
| Non-Psych QOL index | | | .764*** | |  |  | .736*** |  |  | .569*** | | |  |
| EFC Autonomy-promoting | | | .007 | | .110 |  | .203† | .098 |  | .126* | | | .014 |
| EFC Relatedness-promoting | | | -.004 | | .369* |  | .309** | .200† |  | .148* | | | .037 |

*Notes*. Edu 1 = dummy variable representing tertiary educational level; Edu 2 = dummy variable representing PhD educational level; Edu Mo = educational level mother; Edu Fa = educational level father; Overall QOL index = Overall Quality of Life index; EFC Autonomy-promoting = Emotional Fit with Culture in Autonomy-promoting situations; EFC Relatedness-promoting = Emotional Fit with Culture in Relatedness-promoting situations.

† *p* < .10 * *p* ≤ .05 ***p* ≤ .01

Table A2. Results of Hierarchical Linear Regressions predicting Psychological Well-being from Emotional fit with Culture in Positive and Negative Autonomy- and Relatedness-promoting situations across Contexts.

| **Panel A: Study 1:**  **Euro-American cultural context** | | | | | | **Panel B: Study 2:**  **Korean cultural context** | | | | | | | **Panel C: Study 3:**  **Belgian cultural context** | | | | | | | |
| --- | --- | --- | --- | --- | --- | --- | --- | --- | --- | --- | --- | --- | --- | --- | --- | --- | --- | --- | --- | --- |
| **Predictor** | | | | **ΔR²** | **β^a^** | **Predictor** | | | | | **ΔR²** | **β^a^** | **Predictor** | | | | | **ΔR²** | **β^a^** | |
| *Step 1* | | | | .000 |  | *Step 1* | | | | | .034 |  | *Step 1* | | | |  | .015 |  | |
|  | Work-dum | | |  | -.058 |  | Work-dum | | | |  | .208 |  | Valence  Work-dum  Friends-dum | | | |  | .064  .051  -.056 | |
| *Step 2* | | | | .248† |  | *Step 2* | | | | | .129† |  | *Step 2* | | |  | | .015 |  | |
|  | Age  Gender  Class | | |  | .020  -.117  -.405* |  | | Age  Gender  Edu_dum1  Edu_dum2 | | |  | .142  -.049  .028  .096 |  | Age  Gender  Mother edu  Father edu | | | |  | .159**  -.076  .011  .019 | |
| *Step 3* | | | | .387*** |  | *Step 3* | | | | | .419*** |  | *Step 3* | | | |  | .303*** |  | |
|  | Overall QOL | | |  | .880*** |  | | Overall QOL | | |  | .639*** |  | Overall QOL | | | |  | .563*** | |
| *Step 4* | | | | .086 |  | *Step 4* | | | | | .043 |  | *Step 4* | | |  | | .032** |  | |
|  | EFC_Pos_Aut  EFC_Pos_Rel  EFC_Neg_Aut  EFC_Neg_Rel | | |  | .004 -.148  -.349† -.155 |  | | EFC_Pos_Aut  EFC_Pos_Rel  EFC_Neg_Aut  EFC_Neg_Rel | | |  | -.018  .177  -.068  .405** |  | **EFC_Aut**  **EFC_Rel** | | | |  | **.159***  **.133*** | |
| *Step 5* | |  | | .136* |  | *Step 5* | | | |  | .069 |  | *Step 5* | |  | | | .011 |  |  |
|  | Work-dum X EFC_Pos_­Aut  Work-dum X EFC_Pos_Rel  Work-dum X EFC_Neg_­Aut  Work-dum X EFC_Neg_Rel | | | | .283  .187 .305† .169 |  | Work-dum X EFC_Pos_­Aut  Work-dum X EFC_Pos_Rel  Work-dum X EFC_Neg_Aut  Work-dum X EFC_Neg_Rel | | | | | .225  -.215  -.063  -.351* |  | Work-dum X EFC_Aut  Work-dum X EFC Rel  Friends-dum X EFC_Aut  Friends-dum X EFC Rel | | | | |  |  |
|  |  | | | |  |  |  | | | | |  | *Step 6* | | | | | .007 |  |  |
|  |  | | | |  |  |  | | | | |  |  | Valence X EFC_Aut  Valence X EFC Rel | | | | |  |  |
|  |  | | | |  |  |  | | | | |  |  | *Step 7* | | | | .005 |  |  |
|  |  | | | |  |  |  | | | | |  |  | Valence X Work-dum X EFC_­Aut  Valence X Work-dum X EFC_Rel  Valence X Friends-dum X EFC_­Aut  Valence X Friends-dum X EFC_Rel | | | | | |  |
| Total *R²* | | | .893 *(Adj. .785)**** | | | Total *R²* | | | .649 *(Adj. .608)**** | | | | Total *R²* | | | | | .327*** | |  |

*Notes*. Work_dum = Dummy variable representing Work Contexts; Friends_dum = Dummy variable representing Friends Contexts; Family Context is always the reference category; EFC_Aut = Emotional Fit with Culture in Autonomy-promoting situations; EFC_Rel = Emotional Fit with Culture in Relatedness-promoting situations; Edu_1 = dummy variable representing tertiary educational level; Edu_2 = dummy variable representing PhD educational level; Edu_Mother = educational level mother; Edu_Father = educational level father; Overall QOL index = Overall Quality of Life index.

*Adj.* = Adjusted *R².*

^a^ The βs presented here are the ones from the final regression model (i.e. the latest step that significantly contributed to the explained variance).

† † *p* < .15 † *p* < .10 **p* ≤ .05 ***p* ≤ .01 ****p* ≤ .001

Table A3. Results of Hierarchical Linear Regressions predicting Psychological Well-being from Emotional fit with Culture in Autonomy- and Relatedness-promoting situations without controlling for Overall QOL Index. Hypothesized associations appear in bold.

| **Panel A: Study 1:**  **Euro-American cultural context** | | | | | **Panel B: Study 2:**  **Korean cultural context** | | | | | | | | **Panel C: Study 3:**  **Belgian cultural context** | | | | | | | |
| --- | --- | --- | --- | --- | --- | --- | --- | --- | --- | --- | --- | --- | --- | --- | --- | --- | --- | --- | --- | --- |
| **Predictor** | | | **ΔR²** | **β^a^** | **Predictor** | | | | | **ΔR²** | | **β^a^** | **Predictor** | | | | | **ΔR²** | **β^a^** | |
| *Step 1* | | | .001 |  | *Step 1* | | | | | .047† | |  | *Step 1* | |  | | | .006 |  | |
|  | Work-dummy | |  | .159 |  | Work-dummy | | | |  | | .106 |  | Work-dummy  Friends-dummy | | | |  | -.025  -.101 | |
| *Step 2* | | | .245† |  | *Step 2* | | | | | .127† | |  | *Step 2* | | |  | | .016 |  | |
|  | Age  Gender  Class | |  | -.322  -.478*  .074 |  | Age  Gender  Edu_dum1  Edu_dum2 | | | |  | | -.057  -.114  .352**  .458** |  | Age  Gender  Mother edu  Father edu | | | |  | .104  -.030  .025  .065 | |
| *Step 3* | | | .003 |  | *Step 3* | | | | | | .084* |  | *Step 3* | | |  | | .036** |  | |
|  | EFC_Auto  EFC_Rela | |  | -.604†  .023 |  | EFC_Auto  **EFC_Rela** | | |  | | | .168 **.485**** |  | **EFC_Auto**  **EFC_Rela** | | | |  | **.101††**  **.136†** | |
| *Step 4* | |  | .195* |  | *Step 4* | |  | | .045**††** | | |  | *Step 4* | | | |  | .001 |  | |
|  | **Work-dum X EFC_Auto**  Work-dum X EFC Rela | | | **.695***  .126 |  | Work-dum X EFC_Auto  **Work-dum X EFC Rela** | | | | | | -.117 **-.300†** |  | Work-dum X EFC_Auto  Work-dum X EFC Rela  Friends-dum X EFC_Auto  Friends-dum X EFC Rela | | | | |  | |
| Total *R²* | | | .444 *(Adj .222)* † | | Total *R²* | | |  | .303 *(Adj. .195)*** | | | | Total *R²* | | | |  | .060 *(Adj. .009)*† | |  |

*Notes*. Work_dum = Dummy variable representing Work Contexts; Friends_dum = Dummy variable representing Friends Contexts; Family Context is always the reference category; EFC_Aut = Emotional Fit with Culture in Autonomy-promoting situations; EFC_Rel = Emotional Fit with Culture in Relatedness-promoting situations; Edu_1 = dummy variable representing tertiary educational level; Edu_2 = dummy variable representing PhD educational level; Edu_Mother = educational level mother; Edu_Father = educational level father. *Adj.* = Adjusted *R².*

^a^ The βs presented here are the ones from the final regression model (i.e. the latest step that significantly contributed to the explained variance).

† † *p* < .165 † *p*  ≤ .10 **p* ≤ .05 ***p* ≤ .01 ****p* ≤ .001

**Description Extra Analyses on difference between Mean Level intensity of Autonomy and Relatedness-promoting emotions versus Emotional Fit with Culture in Autonomy and Relatedness-promoting situations.**

**General Data Analytic Strategy.**

To test whether the effects were specific to emotional *fit* with culture or to mean intensity ratings of autonomy and relatedness-promoting emotions, we conducted some additional linear regression analyses in which we predicted psychological well-being from both people’s emotional fit with culture and from their mean intensity ratings of four prototypical emotion scales representing both positive and negative autonomy and relatedness-promoting emotions. Again, we controlled for Context (Step 1), Demographic variables (Step 2) and Overall Quality of Life (Step 3). By changing the order of the steps in which we entered the Mean-levels of prototypical emotions and Emotional Fit with Culture as predictors of Psychological Well-being, we tested which type of variable predicted most variation in psychological well-being.

**Study 1: European American sample**

To test whether the effects reported in the manuscript are specific to the *patterning* of emotions in situations that are central to meeting the European American mandate of being autonomous at work, we conducted some follow-up analyses in which we included the *mean intensity* ratings with which European Americans experienced prototypical autonomy and relatedness-promoting emotions. Based on the studies by Kitayama and colleagues (Kitayama et al., 2006) and on the mean intensity levels of all emotions included in Study 1, we selected pride as the most prototypical positive autonomy-promoting emotion (*M* = 3.71, *SD* = .99), closeness as the most prototypical positive relatedness-promoting emotion (*M* = 4.02 *SD* = 1.22), irritation as the most prototypical negative autonomy-promoting emotion (*M* = 3.15, *SD* = .89), and shame as the most prototypical negative relatedness-promoting emotion (*M* = 2.95, *SD* = 1.33). Subsequently, we conducted a hierarchical regression analysis that included the mean levels of the four prototypical emotions (Step 4), after controlling for Context, Demographics and Overall Quality of Life (Steps 1-3). In Step 5, we entered the main effects of EFC and in Step 6, we entered the interactions between EFC and Context.

As reported in Table A4, panel A, only the last step significantly contributed to the prediction of Psychological well-being above the control variables (Step 6, Δ*R²* = .094, *p* = .033); the step with mean intensity ratings was not significant (Step 4: Δ*R²* = .081, *p* = .252). This finding suggests that a) European Americans’ psychological well-being is better predicted by the patterning of emotions in autonomy-promoting situations than by the overall intensity level of prototypical autonomy and relatedness promoting emotions, and that b) the effect of Emotional Fit with Culture holds true even after controlling for mean intensity levels of prototypical emotions. When entering the mean levels of prototypical emotions after the main (Step 4) and interaction effects of EFC and Context (Step 5), the effects of the mean intensity ratings remained non-significant (Step 6: Δ*R²* = .022, *p* = .735).

**Study 2: Korean sample**

To test whether the effects reported in the manuscript are specific to the patterning of emotions in situations that are central to meeting the Korean mandate of being related to family members, we followed the exact same strategy as outlined in Study 1. First, we selected pride (*M* = 3.18, *SD* = .91), closeness (*M* = 3.62 *SD* = 1.00), irritation (*M* = 3.33, *SD* = .76), and shame (*M* = 2.93, *SD* = 1.13) as the most prototypical positive and negative autonomy and relatedness-promoting emotions. Second, we conducted a regression analysis that also included the mean intensity ratings of these four prototypical emotions (Step 4) before testing the effects of EFC (Steps 5 and 6; for the full results, see Table A4, panel B). The regression analysis indicated that the last step, including EFC contributed to the prediction of Psychological well-being (Step 6, Δ*R²* = .026, *p* = .130), whereas the mean intensity ratings did not (Step 4: Δ*R²* = .029, *p* = .334); as before, repeating this analysis by including EFC in Relatedness-promoting situations only, strengthened the results of EFC (Step 6, Δ*R²* = .022, *p* = .056). Entering the mean levels of prototypical emotions as the final step (Step 6), also yielded no significant effects of these mean intensity ratings on Psychological well-being (Step 6: Δ*R²* = .025, *p* = .392). Similar to Study 1, these results thus suggest that a) Koreans’ psychological well-being is better predicted by the patterning of emotions in relatedness-promoting situations than by the overall intensity level of prototypical autonomy and relatedness-promoting emotions, and that b) the above effects of EFC hold true even after controlling for mean intensity levels of prototypical emotions.

**Study 3: Belgian sample**

To test whether the results reported in the manuscript are specific to the patterning of emotions in situations or to the mean intensity levels of specific emotions, we ran additional regression analyses. Because the Principal Component Analysis on the emotion data had yielded a clear four factor structure referring to positive and negative autonomy and relatedness-promoting emotions, we decided to make use of these emotion scales rather than of single prototypical emotions. All scales had good reliability in both the first and second situation: positive autonomy-promoting scale (relieved, proud about myself, euphoric, surprised, hopeful, strong; α = .87 and .87 for the first and second situation, respectively; *M =* 3.28, *SD* = 1.48 averaged across both situations), positive relatedness-promoting scale (relying, close, respect, proud about another, helpful, interested, grateful; α = .90, 85; *M =* 3.45, *SD* = 1.43), negative autonomy-promoting scale (angry, disappointed, irritated, offended, frustrated, upset, ill feelings, sad, depressed; α = .95, .93; *M =*2.89, *SD* = 1.61), and negative relatedness-promoting scale (indebt, ashamed, guilty, embarrassed, worthless, afraid; α = .85, .84; *M =* 2.62, *SD* = 1.30).

Unlike Studies 1 and 2, the regression analysis indicated that the step with mean intensity ratings significantly contributed to the prediction of Belgians’ psychological well-being (Step 4: Δ*R²* = .033, *p* = .024; see Table A4, panel C). Step 5, which included the main effects of EFC was still marginally significant above and beyond these mean-level effects (Step 5: Δ*R²* = .016, *p* = .067), although the effects of EFC on psychological well-being were weaker than before (β_EFC_Autonomy_ = .112, *p* = .080; β_EFC_Relatedness_ = .096, *p* = .160). The mean intensity of negative relatedness-promoting emotions was the only (marginally) significant effect of the mean intensity ratings (β_Neg_Rela_ = -.164, *p* = .085), with more feelings of shame, guilt, indebtedness, etc., being associated with lower levels of Psychological Well-being. When reversing the order of the steps – i.e. when entering the main effects of EFC in step 4, their interactions with the Contexts in step 5 and the mean level variables in step 6 – the mean level intensity ratings did no longer contribute to the explanation of Psychological Well-being (Step 6: Δ*R²* = .013, *p* = .330), suggesting that EFC may explain some variance above and beyond mean level effects of individual emotion scales, whereas the opposite is not true.

Table A4. Results of Hierarchical Linear Regressions predicting Psychological Well-being from Emotional fit with Culture in Autonomy- and Relatedness-promoting situations in Home, Work and Friend contexts, after controlling for the effects of mean intensity scores for Positive and Negative Autonomy and Relatedness-promoting emotions.

| **Panel A: Study 1:**  **Euro-American cultural context** | | | | | | **Panel B: Study 2:**  **Korean cultural context** | | | | | | | | **Panel C: Study 3:**  **Belgian cultural context** | | | | | | | | |
| --- | --- | --- | --- | --- | --- | --- | --- | --- | --- | --- | --- | --- | --- | --- | --- | --- | --- | --- | --- | --- | --- | --- |
| **Predictor** | | | **ΔR²** | | **β^a^** | **Predictor** | | | | | | **ΔR²** | **β^a^** | **Predictor** | | | | | | **ΔR²** | **β^a^** | |
| *Step 1* | | | .000 | |  | *Step 1* | | | | | | .047† |  | *Step 1* | | | |  | | .006 |  | |
|  | Work-dum | |  | | -.047 |  | Work-dum | | | | |  | .185* |  | Work-dum  Friends-dum | | | | |  | .055  -.071 | |
| *Step 2* | | | .245† | |  | *Step 2* | | | | | | .127† |  | *Step 2* | | |  | | | .016 |  | |
|  | Age  Gender  Class | |  | | -.001  -.271†  -.258† |  | | Age  Gender  Edu dum1  Edu dum2 | | | |  | .181  -.050  -.026  -.043 |  | Age  Gender  Mother edu  Father edu | | | | |  | .151**  -.071  -.003  .026 | |
| *Step 3* | | | .409*** | |  | *Step 3* | | | | | | .449*** |  | *Step 3* | | | |  | | .303*** |  | |
|  | Overall QOL | |  | | .762*** |  | | Overall QOL | | | |  | .679*** |  | Overall QOL | | | | |  | .551*** | |
| *Step 4* | | | .081 | |  | *Step 4* | | | | | | .029 |  | *Step 4* | | |  | | | .033* |  | |
|  | Mean Pride  Mean Closeness  Mean Irritation  Mean Shame | | |  | -.076  .136  .124 -.126 |  | | Mean Pride  Mean Closeness  Mean Irritation  Mean Shame | | | |  | .092  .121  -.031  -.139 |  | Mean Pos Auto  Mean Pos Rela  Mean Neg Auto  Mean Neg Rela | | | | |  | .085  -.029  .164  -.164† | |
| *Step 5* |  | | .007 | |  | *Step 5* | | | | |  | .002 |  | *Step 5* | |  | | | | .016† |  |  |
|  | EFC Auto  EFC Rela | | | | -.381†  -.201 |  | EFC_Auto  EFC_Rela | | | | | | -.108  .252† |  | EFC_Auto  EFC_Rela | | | | | | .112†  .096 |  |
| *Step 6* |  | | .094* | |  | *Step 6* | | |  | | | .026†† |  | *Step 6* | | | | | | .012 |  |  |
|  | Work-dum X EFC_Auto  Work-dum X EFC Rela | | | | .646*  .106 |  | Work-dum X EFC_Auto  Work-dum X EFC Rela | | | | | | .127  -.267* |  | Work-dum X EFC_Auto  Work-dum X EFC Rela  Friends-dum X EFC_Auto  Friends-dum X EFC Rela | | | | | |  |  |
| Total *R²* | | .873 *(Adj. .696)**** | | | | Total *R²* | | | | .679 *(Adj. 595)**** | | | | Total *R²* | | | | | .375*(Adj. 338)**** | | |  |

*Notes*. Work-dum = Dummy variable representing Work Contexts; Friends-dum = Dummy variable representing Friends Contexts; Family Context is always the reference category; Edu dum1 = dummy variable representing tertiary educational level; Edu dum2 = dummy variable representing PhD educational level; Edu Mother = educational level mother; Edu Father = educational level father; Overall QOL index = Overall Quality of Life index; Mean Pos Auto = Mean intensity Positive Autonomy-promoting emotions; Mean Pos Rela = Mean intensity Positive Relatedness-promoting emotions; Mean Neg Auto = Mean intensity Negative Autonomy-promoting emotions; Mean Neg Rela = Mean intensity Negative Relatedness-promoting emotions; EFC Auto = Emotional Fit with Culture in Autonomy-promoting situations; EFC Rela = Emotional Fit with Culture in Relatedness-promoting situations; *Adj.* = Adjusted *R².*

^a^ The βs presented here are the ones from the final regression model (i.e., the latest step that significantly contributed to the explained variance).

† † *p* < .15 † *p* < .10 **p* ≤ .05 ***p* ≤ .01 ****p* ≤ .001
